# Porcine Brain Enzyme Hydrolysate Enhances Immune Function and Antioxidant Defense via Modulation of Gut Microbiota in a Cyclophosphamide-Induced Immunodeficiency Model

**DOI:** 10.3390/antiox13040476

**Published:** 2024-04-17

**Authors:** Yu Yue, Hye Jeong Yang, Ting Zhang, Chen Li, Min Jung Kim, Keun-Nam Kim, Sunmin Park

**Affiliations:** 1 Department of Food and Nutrition, Obesity/Diabetes Research Center, Hoseo University, Asan 31499, Republic of Korea; 20193142@vision.hoseo.edu (Y.Y.); zhangting92925@gmail.com (T.Z.); lic77732@gmail.com (C.L.); 2 Food Functionality Research Division, Korea Food Research Institute, Wanju 55365, Republic of Korea; yhj@kfri.re.kr (H.J.Y.); kmj@kfri.re.kr (M.J.K.); 3 Department of R&D, UNIMED PHARM Inc., Seoul 05567, Republic of Korea; dudgns1225@unimed.co.kr

**Keywords:** porcine brain enzyme hydrolysate, immune function, natural killer cell activity, splenocyte proliferation, Th1/Th2 ratio, gut microbiota

## Abstract

This study examined how consuming porcine brain enzyme hydrolysate (PBEH) affects the immune function and composition of the gut microbiota in an immunodeficient animal model. Male Wistar rats aged 6 weeks were fed casein (control), 100 mg/kg body weight (BW), red ginseng extract (positive-control), and 6, 13, and 26 mg PBEH per kg BW (PBEH-L, PBEH-M, and PBEH-H, respectively) daily for 4 weeks. At 30 min after consuming assigned compounds, they were orally administered cyclophosphamide (CTX; 5 mg/kg BW), an immunosuppressive agent, to suppress the immune system by inhibiting the proliferation of lymphocytes. The normal-control rats were fed casein and water instead of CTX. Natural killer cell activity and splenocyte proliferation induced by 1 μg/mL lipopolysaccharide were lower in the control group than the normal-control group, and they significantly increased with PBEH consumption, particularly at high doses. The PBEH consumption increased dose-dependently in the Th1/Th2 ratio compared to the control. The lipid peroxide contents were lower in the PBEH group than in the control group. Moreover, PBEH m and PBEH-H consumption mitigated white pulp cell damage, reduced red pulp congestion, and increased spleen mast cells in the histological analysis. Intestinal microbiota composition demonstrated differences between the groups at the genus levels, with *Akkermansia* being more abundant in the control group than the normal-control group and the PBEH-H group showing a decrease. However, *Bifidobacterium* decreased in the control group but increased in the PBEH-H group. The β-diversity revealed distinct microbial communities of PBEH and positive-control groups compared to the control group (*p* < 0.05). The metagenome predictions revealed that PBEH-H influenced amino acid metabolism, antioxidant defense, insulin sensitivity, and longevity pathways. In conclusion, PBEH-H intake boosted immune responses and reduced lipid peroxides by modulating gut microbiota composition. These findings suggest that PBEH-H has the potential as a dietary supplement for improving immune function and gut health in individuals with immunodeficiency.

## 1. Introduction

The immune system is a complex biological network that defends the body against infections. It is classified into two subsystems: the innate immune system, which acts as the initial, immediate, and non-specific response against pathogens, followed by the activation of the adaptive immune system, the second layer of protection [1]. Adaptive immunity, characterized by immunological memory, enables a quicker and more robust elimination of specific pathogens. Immunodeficiency arises from the malfunctioning or lack of crucial elements within the immune system, such as lymphocytes, phagocytes, and the complement system [1]. The immune system’s responsiveness to pathogens is diminished in older adults, and its decline typically commences around the age of 50, a phenomenon known as immunosenescence [2]. Malnutrition, obesity, alcoholism, and drug abuse can contribute to immune dysfunction [3].

The immune system interacts intricately with the endocrine system, central nervous system (CNS), and autonomic nervous system (ANS), playing a crucial role in tissue repair and regeneration [4]. Hormones such as sex hormones, prolactin, growth hormone, and vitamin D actively regulate the immune system. When the CNS experiences an injury, the delicate balance between the immune system and the CNS is disrupted [5]. CNS injury suppresses cell-mediated immune responses via the hypothalamic–pituitary–adrenal axis and the sympathetic and parasympathetic nervous systems [5]. The ANS governing innate and adaptive immunity can influence inflammatory responses, and an imbalance in this system may lead to an altered inflammatory response [4]. Moreover, dysfunction in the ANS is closely associated with increased inflammation and cardiovascular risk. The ANS also interacts with the gut microbiota to maintain an optimal inflammation state [4].

The gut microbiota actively regulates the immune response via intricate crosstalk with both innate and adaptive immune systems [6]. Under normal conditions, the host’s immune response to the intestinal microbiota is precisely compartmentalized to the mucosal surface. Fundamental structures such as the mucosal barrier and tight junctions are critical in restricting trans-epithelial permeability, thereby achieving compartmentalization of the microbiota [7]. Gut dysbiosis can result from various factors, including infection, inflammation, dietary choices, exposure to xenobiotics, host genetics, or the host’s environment [8]. A dysbiosis potentially influences the colonization niche of harmful bacteria, contributing to the alteration in innate and adaptive intestinal immunity.

Consuming porcine brain enzyme hydrolysate (PBEH) has been reported to enhance memory function in rats with scopolamine-induced memory decline. This improvement is attributed to the modulation of the parasympathetic nervous system [7]. This improvement has been attributed to PBEH-associated reduction in neuroinflammation and insulin resistance [7]. Interestingly, PBEH intake in the earlier study was linked to enhanced gut dysbiosis, characterized by an elevation in *Clostridium* and a reduction in *Bifidobacterium* [7]. Additionally, PBEH has antioxidant properties and has been reported to reduce oxidative stress and inflammation [9]. Red ginseng extract has been shown to enhance immune responses, improve immune function, and reduce oxidative stress in immunocompromised animals [9]. PBEH, including its reported antioxidant capacity, may provide distinct advantages over red ginseng extract in modulating immunity and oxidative stress. Therefore, red ginseng extract can serve as a reference material for assessing the efficacy of PBEH as a novel immunomodulatory agent, with its potential antioxidant effects synergistically enhancing its immunomodulatory properties. Thus, the present study aimed to investigate the immunomodulatory effects of PBEH intake in cyclophosphamide (CTX)-induced immune-deficient rats while exploring its associated mechanisms. The study sought to understand the potential immunomodulatory mechanisms associated with PBEH in reducing oxidative stress.

## 2. Methods

### 2.1. Production of PBEH

Fresh porcine brains were weighed and thawed. A series of processing steps were carried out to obtain PBEH by enzymatic hydrolysate of the porcine brain. First, the brains were washed sequentially with distilled water to remove blood, followed by grinding. Ethanol was used twice to remove lipids, and subsequently, a heating process was performed to remove ethanol and effectively denature proteins to eliminate steroid and peptide hormones. The hydrolysis stage involved the digestion of porcine brain proteins with proteases, followed by enzyme inactivation via heating and filtration. Undigested proteins were precipitated with fermented ethanol, filtered using a microfiltration method, and concentrated. Finally, sterilization was performed, followed by freeze-drying, resulting in a yield of about 5% relative to the initial weight of fresh porcine brains. It was generously provided by Ubio (Seoul, Republic of Korea). The PBEH mainly contained 2–5 kD peptides; its primary amino acid constituents were arginine, lysine, leucine, and phenylalanine (Supplementary Table S1). The primary amino acids in casein used for the control group were glutamic acid, proline, leucine, and lysine, but they did not contain small peptides. For the positive control, red ginseng water extracts (1 g) contained 13.1 mg of ginsenosides, including 3.3 mg of Rg1, 5.8 mg of Rb1, 2.3 mg of Rb2, and 1.7 mg of Rc, along with 0.4 g of sugar and soluble dietary fiber (Korea Ginseng Corporation, Daejeon, Republic of Korea) [10]. PBEH and red ginseng were utilized in the animal feeding study.

### 2.2. Animal Care and Immunosuppressive Animal Model Induced using CTX

Male Wistar rats aged 5 weeks and grown in a specific pathogen-free (SPF) state were purchased from Samtako Bio Korea (Osan, Republic of Korea). Throughout the acclimatization and experimental phases, the rats were given ad libitum access to standard pellets designed for growing rats (Samtako, Gyeonggi, Republic of Korea). They were given filtered water replenished daily under controlled conditions [7]. Environmental conditions were carefully regulated, maintaining a temperature of 23 ± 1 °C, humidity at 50 ± 5%, noise levels below 60 decibels, 12 h lighting cycle from 08:00 to 20:00, illumination ranging from 150 to 300 Lux, and ventilation set at 10–12 changes per hour. The animal study was approved by the Institutional Animal Care and Use Committee of Hoseo University (HSIACUC-17-071), and it followed the Guide for the Care and Use of Laboratory Animals (8th edition) issued by the National Institutes of Health.

CTX, an immunosuppressive agent, was obtained from Sigma-Aldrich (St. Louis, MI, USA) and dissolved in distilled water. Based on the preliminary research findings, the suitable CTX oral administration dosage was 5 mg/kg BW. The rats were randomly divided into six groups (*n* = 10) as follows: normal-control (no CTX administration and 26 mg casein/kg BW), control (CTX administration and 26 mg casein/kg BW), low-PBEH (PBEH-L, CTX administration and 6 mg PBEH/kg BW), medium-PBEH (PBEH-M, CTX administration and 13 mg PBEH/kg BW), high-PBEH (PBEH-H, CTX administration and 26 mg PBEH/kg BW), or positive-control (CTX administration and 100 mg red ginseng extract/kg BW). Each day, the rats were administered their assigned treatment (water, PBEH, or red ginseng extract), followed by CTX provided 30 min later. This sequence was repeated daily for 4 weeks, with the assigned extracts and CTX administered via oral gavage. Body weight and food consumption were assessed weekly at 10 am on Tuesdays.

Twenty-four hours later, after the completion of their last administration, rats had anesthetization with ketamine and xylazine mixture (100 and 10 mg/kg BW). Blood samples were collected from the rats via the vena cava and portal vein, using both EDTA-treated and non-EDTA tubes. Subsequently, the spleen and thymus were surgically dissected from the animals. The spleen index and thymus index were calculated by dividing the respective organ weight (spleen or thymus) by the final body weight of the rat at the end of the intervention period. After anesthetizing the rats with ketamine and xylazine (100 and 10 mg/kg BW), the splenocytes were isolated from 6 rats per each group to measure the proliferation of splenic cells. The natural killer (NK) cell activity was determined in the splenocytes. The WBC count of the EDTA-treated peripheral blood was measured using a semi-automatic blood cell analyzer (Hemavet 950Fs, Drew Scientific Inc., Plantation, FL, USA) using the automatic standard method.

Interferon-γ (IFN-γ), interleukin (IL)-1β, IL-2, and IL-4 concentrations in the serum were assessed using enzyme-linked immunosorbent assay (ELISA) kits from Thermo Fisher Scientific (Waltham, MA, USA), including the IFN gamma Rat ELISA Kit, IL-1 beta Rat ELISA Kit, IL-2 Rat ELISA Kit, and Rat IL-4 ELISA Kit. Serum immunoglobulin (Ig)A, IgG, and IgM concentrations were measured using the IgA Rat Uncoated ELISA Kit, IgG (Total) Rat Uncoated ELISA Kit, and IgM Rat Uncoated ELISA Kit, respectively, from Thermo Fisher Scientific. Additionally, serum alanine aminotransferase (ALT), aspartate aminotransferase (AST), and glucose concentrations were measured using the Asan set GPT Assay kit, Asan set GOT Assay kit, and Glucose Assay kit (Asan Pharmaceutics, Seoul, Republic of Korea), respectively, with a spectrophotometer. The spleen and large intestines of the remaining four rats per each group were dissected. Lipid peroxide contents were measured with a Lipid Peroxidation (MDA) assay kit (Abcam, Cambridge, UK).

### 2.3. Isolation of Splenocytes and Their Proliferation Induced using Lipopolysaccharides (LPS)

Splenocytes were aseptically harvested from the spleen dissected from the rats, rinsed with RPMI-1640 medium (Thermo Fisher Scientific, Waltham, MA, USA), and subsequently minced lightly with a sterilized glass rod. The dispersed cell suspension was passed through a 200 μm-mesh stainless steel sieve, washed twice with RPMI media containing 10% fetal bovine serum, and centrifuged at 1000 rpm for 5 min. The splenocytes were adjusted to a 3.0 × 10^6^ cells/mL concentration in the culture medium. The cell suspension was dispensed into each well of a 96-well plate (Nunc, Roskilde, Denmark), and LPS (Sigma-Aldrich, St. Loise, MO, USA) was added to achieve the final 1 µg/mL concentrations. An equal volume of culture medium was dispensed in the control group. The cells were then incubated at 37 °C in a 5% CO_2_ incubator. After 48 h, water-soluble tetrazolium (WST, Sigma-Aldrich) was added to each well at 10% of the volume and incubated for 2 h. The optical density (OD) was measured at 405 nm using a microplate reader (Molecular Devices, San Jose, CA, USA) to compare the cell activity. The cell proliferation rate was calculated using the formula: Cell proliferation assay (%) = (Absorbance of the sample-treated group/Absorbance of the control group) × 100 [11].

After 4 h, splenic cells, isolated and concentrated at 4 × 10^6^ cells per well, were introduced into a 48-well plate. Subsequently, they were treated with LPS at a concentration of 1 μg/mL. The cells were cultured for 24 h at 37 °C with 5% CO_2_. After incubation, the culture supernatant was collected, and the levels of IFN-γ, IL-2, IL-4, IL-6, and IL-10 were measured using ELISA kits.

### 2.4. Natural Killer Cell Activity

The effector cells, isolated splenic cells, were plated at 5 × 10^6^ cells/mL in a 96-well plate along with the target cells, AR42J cells, at various effector-to-target cell ratios (1:20) [12]. The co-culture was maintained in a 37 °C, 5% CO_2_ incubator for 24 h. At the post-culture, lactate dehydrogenase (LDH) measurement was performed using the LDH cytotoxicity detection kit (Takara Bio, Shiga, Japan). The absorbance of formazan, generated by the oxidation of nicotinamide adenine dinucleotide in the reaction, was measured at 490 nm for comparison and analysis against the control group.

### 2.5. Histology of the Spleen and Large Intestines

The dissected spleen and large intestines were first perfused sequentially with saline, followed by a 4% paraformaldehyde solution (pH 7.2). Subsequently, the perfused tissues were embedded in paraffin [13]. Two random 5-μm paraffin-embedded spleen and large intestine sections were selected and stained with hematoxylin and eosin (H&E). Additionally, toluidine blue staining was performed on the spleen sections to analyze mast cells. Alcian blue-perchloric acid (PAS) staining was employed on the large intestine section to assess mucin levels [14].

The H&E-stained spleen sections were observed using a Zeiss Axiovert microscope with a DIXI Imaging tool at 10× magnification. It allowed for the examination of various parameters, including cell damage, cell aggregation, and the presence of surviving cells within the white pulp of the spleen section, as well as the assessment of congestion within the red pulp. The number of mast cells was calculated from the toluidine-stained spleen section [9].

In the H&E-stained section of the intestinal villi, measurements were taken for the length and width of the large intestine villi and the height of the crypts. Additionally, impaired cells were counted, and their relative area was scored on a scale from 0 to 3 as follows: 0 (no or minimal impairment) indicates no or very few impaired cells observed; 1 (mild impairment) indicates a small area of the villi or crypt showing impaired cells, affecting up to 25% of the observed area; 2 (moderate impairment) indicates a moderate area of impaired cells, affecting between 25% and 50% of the observed area; 3 (severe impairment) indicates a large area of impaired cells, affecting more than 50% of the observed area, with extensive damage or disruption of the villi or crypt structure [15]. Furthermore, the percentage of intestinal goblet cells producing mucin, as indicated using blue staining in the Alcian blue-PAS-stained sections, was calculated.

### 2.6. Serum Short-Chain Fatty Acid (SCFA) Concentrations and Gut Microbiome

To investigate the potential association between gut microbial metabolism and host immunity, serum SCFA concentrations were assessed [16]. Given the systemic nature of SCFAs and their role in mediating various physiological processes beyond the gastrointestinal tract, we measured SCFA levels in the portal vein blood. This approach allows for evaluating SCFA absorption into the bloodstream, providing insights into their systemic distribution and potential impact on host health. Portal vein blood samples were collected and processed to obtain serum, which was then mixed with ethanol (Duksan, Ansan, Korea) and 1N hydrochloric acid before centrifugation at 15,000 rpm for 15 min at 4 °C. The supernatants underwent gas chromatography analysis utilizing a Clarus 680 GAS chromatograph (Perkin Elmer, Waltham, MA, USA) equipped with an Elite-Free Fatty Acid Phase capillary column. Helium served as the carrier gas, flowing at a rate of 1 mL/min [17]. Calibration was performed using external standards of acetate, propionate, and butyrate (1 mM; Sigma-Aldrich). 

Metagenome sequencing employing next-generation sequencing (NGS) techniques was utilized to explore the microbiota communities originating from each rat’s cecum [7]. Fecal bacterial DNA extraction was conducted according to the manufacturer’s protocol using the Power Water DNA Isolation Kit (Qiagen, Valencia, CA, USA). Subsequently, polymerase chain reaction (PCR) amplification was performed using 16S amplicon primers, and libraries were prepared for the PCR products per the genome sequencer FLX plus library prep guide, as previously described [17]. PCR amplification was carried out using 16S universal primers in the FastStart High-Fidelity PCR System (Roche, Basel, Switzerland), following the manufacturer’s guidelines. The extracted bacterial DNA from cecal feces underwent sequencing using the Illumina MiSeq standard operating procedure and a Genome Sequencer FLX plus (454 Life Sciences) at Macrogen (Seoul, Republic of Korea).

The 16S amplicon sequences obtained were processed utilizing the Mothur v.1.36 package. Fecal bacterial taxonomy was assigned following the Miseq SOP, and bacterial counts were performed for each fecal sample. Sequence alignment was conducted using the Silva reference alignment v.12350, with bacterial counts and identifications performed as described previously [17]. The relative bacterial counts were computed in the taxonomic assignment order for each sample. Additionally, the α-diversity was assessed using Shannon and Chao1 indices. Principal Coordinates Analysis (PCoA) results for gut bacteria were visualized using the R package software version 3.3.0. Multiple comparisons among the groups were analyzed using Permanova at a significance level of *p* < 0.05.

### 2.7. Metagenome Functions Related to Metabolism using PICRUSt2 Pipeline Analysis

The metabolic functions of the gut microbiota were analyzed using metagenomic analysis. Prediction of metabolic pathways was performed using the Phylogenetic Investigation of Communities by Reconstruction of Unobserved States 2 (PICRUSt2) software version 2.5.2 [18]. This analysis utilized the FASTA files and count tables of fecal bacteria to predict functional pathways. The Kyoto Encyclopedia of Genes and Genomes (KEGG) Orthologues (KO) mapped by the KEGG mapper employed for pathway prediction [13]. These analyses aimed to uncover variations in metabolic functions among the groups based on the composition of the gut microbiome.

### 2.8. Statistical Analysis

Statistical analysis was conducted using SAS software version 7 (SAS Institute, Cary, NC, USA). The optimal sample size was determined using the G power program version 3.1 (power = 0.90 and effect size = 0.5), resulting in a calculated sample size of 10 per group. Data were presented as means ± standard deviation (SD) for normally distributed data, as confirmed using the Proc univariate procedure. One-way analysis of variance (ANOVA) was employed for data analysis. Tukey’s test assessed significant differences among the groups, with significance set at *p* < 0.05.

## 3. Results

### 3.1. Body Weight and Immunity-Related Organ Weight Changes

Throughout the experimental period, the normal-control group exhibited a significant weekly increase in body weight compared to the control group, with the PBEH groups showing a less pronounced increase relative to the normal-control group (Figure S1). Compared to the control, significant weight gain was observed in the PBEH groups (Table 1). Immunocompromised animals treated with CTX displayed a reduced dietary intake, while the PBEH groups exhibited an increase in dietary intake similar to the normal-control group (Table 1). Indices reflecting immune function, such as spleen and thymus indexes, significantly decreased in the control group compared to the normal-control group. Although there was a dose-dependent rise in the spleen index in the PBEH groups relative to the control group, statistical significance was only achieved in the PBEH-H group. However, the increase observed in the PBEH-H group did not reach statistical significance compared to the normal-control group. The thymus index was notably higher in the normal-control group than in the control group, but no significant difference was observed between the control and PBEH groups (Table 1).

### 3.2. Liver Damage Index

Liver damage indicators, including serum ALT and AST concentrations, were elevated in the control group compared to the normal-control group (Table 1). The PBEH groups exhibited lower levels of serum ALT concentration than the control group, and the PBEH-Mand positive-control groups showed a decrease in levels equivalent to the normal-control group. However, only the PBEH-M group showed a decrease in serum AST concentrations compared to the control group, but it was much higher than the normal-control group (Table 1). The lipid peroxide contents in the liver were higher in the control group than in the normal-control group and decreased with PBEH dose-dependent. The contents were similar in the PBEH-H group to the normal-control group.

### 3.3. WBC Count and Its Components

The WBC count exhibited a significant decrease (2.5-fold) in the control group compared to the normal-control group. However, no statistically significant difference is observed for control, PBEH, and positive-control (Table 2). WBC components, including lymphocytes, granulocytes, and MID, were quantified to assess immune function. The lymphocyte count was notably lower in the control group compared to the normal-control group. However, it demonstrated an increase in both the positive-control and PBEH-H groups relative to the control group, albeit not reaching the levels observed in the normal-control group (Table 2). Granulocyte and MID counts were significantly diminished in the control group compared to the normal-control group, with the PBEH and positive-control groups displaying values similar to those in the control group (Table 2).

### 3.4. Splenocyte Proliferation and NK Cell Activity

In the absence of LPS-induced inflammation, the spleen cell proliferation decreased by approximately 20% in the control group compared to the normal-control group. However, PBEH exhibited a dose-dependent increase, resulting in spleen cell proliferation rates similar to those of the positive-control and normal-control groups. Nevertheless, these differences did not reach statistical significance (Table 3).

Upon LPS stimulation of the spleen cells, their proliferation rates lowered by about 28% in the control group than in the normal-control group. The PBEH and positive-control groups showed a statistically significant increase in spleen cell proliferation compared to the control group (Table 3). Notably, the PBEH-H and positive-control groups showed an increase in the proliferation rate as much as the normal-control (Table 3).

NK cell activity in the normal-control group decreased by approximately 50% compared to the control group. However, the activity was PBEH (73–93.6%) and positive-control (77%) groups based on the normal-control, and the PBEH-H group (93.6%) surpassed the positive-control group and approached levels similar to those of the normal-control group (Table 3).

Lipid peroxide levels in the splenocytes were elevated in the control group compared to the normal-control group and exhibited a dose-dependent decrease with PBEH administration (Table 3). Specifically, the lipid peroxide contents were comparable between the PBEH m group and the normal-control group, while they were lower in the PBEH-H group compared to the normal-control group (Table 3).

### 3.5. Spleen Morphology

**Figure 1:**
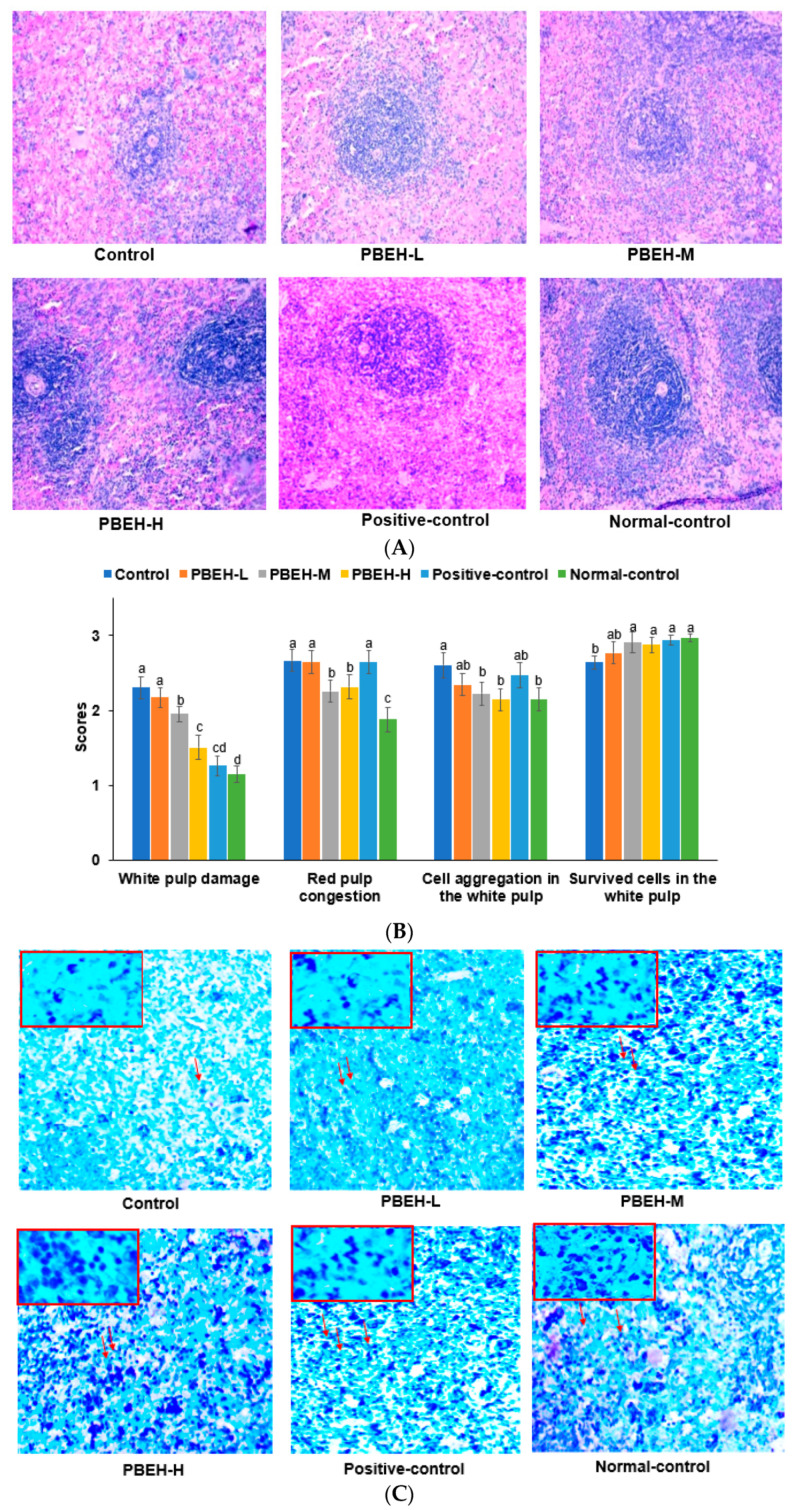

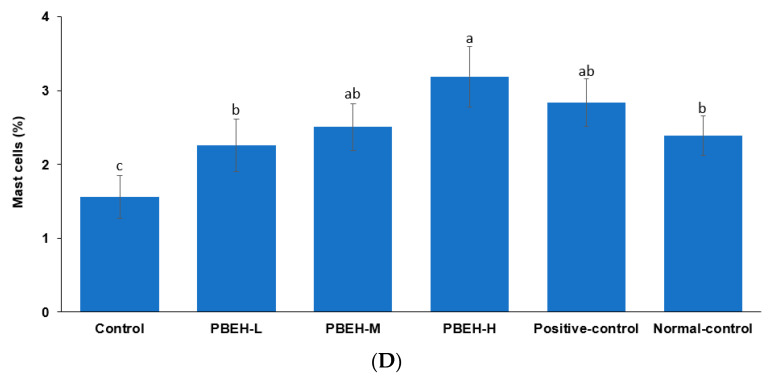
The spleen morphology Scores of spleen cell damage and congestion in the white and red pulp. Bars and error bars are presented as means ± standard deviation (*n* = 10). (**A**) Hematoxylin-eosin staining in the spleen (magnification ×100); (**B**) White pulp cell damage and aggregation and red pulp cell congestion; (**C**) Toluidine staining in the spleen (magnification ×100 in large image and ×400 in small image of the red box); (**D**) Mast cell contents in the spleen. A red arrow indicates the mast cell. Different superscripts (a, b, c) denote significant group differences using Tukey’s test at *p* < 0.05.

In the H&E stained spleen sections, the damage scores of cells within the white pulp region, responsible for producing white blood cells and antibodies crucial for infection protection, were significantly higher in the control group compared to the normal-control group (Figure 1A,B), indicating greater damage within the white pulp. The PBEH groups displayed a dose-dependent reduction in damage scores, with levels similar to those of the positive-control group. However, even in the PBEH-H group, the reduction was not as pronounced as in the normal-control groups (Figure 1A,B). Similarly, congestion within the red pulp region, responsible for removing damaged cells and waste materials, was notably elevated in the control group compared to the normal-control group, suggesting increased congestion within the red pulp area. Although the positive-control group did not alleviate congestion in the red pulp area, the PBEH m and PBEH-H groups exhibited reduced scores, indicating a potential alleviation of congestion within the red pulp region (Figure 1A,B).

The mast cell content in the white pulp region of the spleen was diminished in the control group compared to the normal-control group (Figure 1C,D). The PBEH, positive-control, and normal-control groups showed increased mast cell content compared to the control group, and the PBEH-H group had a higher content than the normal-control (Figure 1C,D).

### 3.6. Serum immunoglobulin and Cytokine Cincentrationss

Serum immunoglobulin levels, including IgA, IgG, and IgM, were lower in the control group than in the normal-control group (Table 4). Serum IgA levels in the positive-control and PBEH-H groups were higher than those in the control group but lower than those in the normal-control group. The PBEH-M, PBEH-H, and positive-control groups exhibited higher IgG levels than the control group, showing values similar to the normal-control group (Table 4). The serum IgM levels showed a similar trend to IgG, with higher levels in the positive-control and PBEH-H groups compared to the control group but lower than the normal-control group (Table 4).

There was no significant difference in IL-1β levels between the normal-control and control groups. In contrast, the PBEH-M, PBEH-H, and the positive-control groups exhibited lower levels than the control group (Table 4). IL-4 levels were higher in the control group than the normal-control group, with levels in the PBEH and positive-control groups similar to those in the control group (Table 4). Similarly, IL-6 levels were lower in the control group than in the normal-control group. In comparison, higher levels were observed in the PBEH-M group, and the positive-control group displayed lower levels than the control group. IFN-γ levels were lower in the control group than the normal-control group, with the PBEH-M and positive-control groups showing higher levels than the control group (Table 4). The T-helper (Th)1/Th2 ratio calculated with the IFN-γ and IL-4 contents ratio was higher in the normal-control group than the control group, indicative of a decreased immune response to pathogens and stimulation of an autoimmune response in the control group. This ratio was also higher in the PBEH-M and PBEH-H groups than in the control group but lower than in the normal-control group.

### 3.7. Cytokine Contents in the Splenocytes

**Figure 2:**
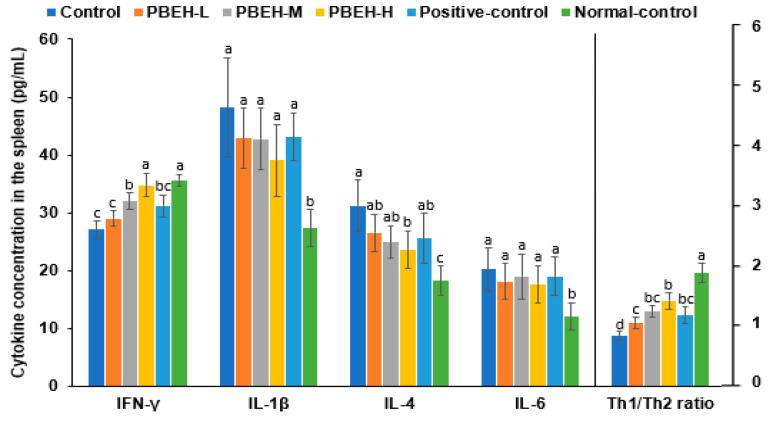
Cytokine concentration in the splenocytes induced by lipopolysaccharide. Bars and error bars are presented as means ± standard deviation (*n* = 10). Different superscripts (a, b, c) denote significant group differences using Tukey’s test at *p* < 0.05.

In the spleen, the IFN-γ contents were higher in the normal-control group compared to the control group and demonstrated a dose-dependent increase in the PBEH group. Notably, the PBEH-H group exhibited IFN-γ levels similar to the normal-control group. Conversely, the IL-1β and IL-6 contents in the spleen were lower in the normal-control group compared to the control group, with no significant difference observed between the PBEH and positive-control groups relative to the control group (Figure 2). The IL-4 content was higher in the control group than in the normal-control group. It was lower in the PBEH-H group than the control group but higher than the normal-control group. Moreover, the Th1/Th2 ratio calculated with the ratio of IFN-γ and IL-4 contents was notably higher in the normal-control group compared to the control group, with the PBEH-H group showing a higher ratio than the control group but lower than that of the normal-control group (Figure 2).

### 3.8. Large Intestine Morphology

In the large intestines, the villi length was observed to be shorter in the control group compared to the normal-control group. In contrast, both the PBEH-M and PBEH-H groups exhibited longer villi lengths comparable to those of the normal-control group (Figure S2A,B). Conversely, the villi width appeared wider in the control group and demonstrated a decrease in both the PBEH-H and positive-control groups. Additionally, the crypt height was found to be shorter in the control group relative to the normal-control group, with the PBEH-H group displaying a height similar to that of the normal-control group (Figure S2A,B). Furthermore, the contents of mucin-producing cells and goblet cells were lower in the control group compared to the normal-control group. However, there was an increase in the contents in the PBEH group compared to the control group, with both the PBEH-M and PBEH-H groups exhibiting goblet cells similar to those of the normal-control group (Figure S2C,D).

### 3.9. SCFAs in the Portal Vein and Gut Microbiota in the cecum

**Figure 3:**
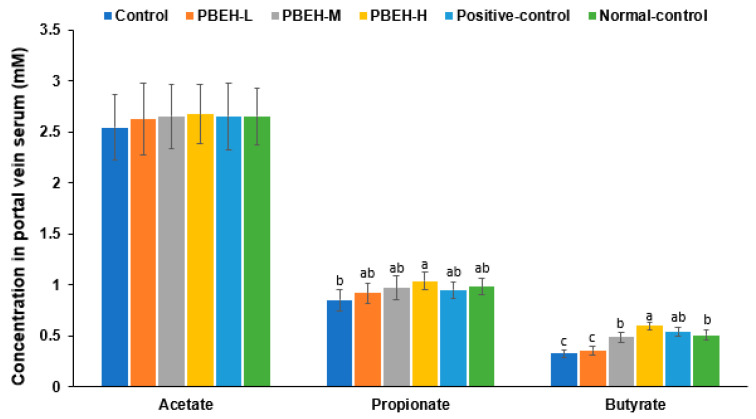
Short-chain fatty acid (SCFA) concentrations in the portal vein. Bars and error bars are presented as means ± standard deviation (*n* = 10). Different superscripts (a, b, c) denote significant group differences using Tukey’s test at *p* < 0.05.

There were no significant differences in serum acetate concentrations among the groups, while serum propionate concentrations were higher only in the PBEH-H group compared to the control group. Serum butyrate concentration was lower in the control group compared to the normal-control group and higher in the PBEH-M, PBEH-H, and positive-control groups compared to the control group (Figure 3). Specifically, the serum butyrate concentration in the PBEH-H group was higher than in the normal-control group.

At the family level, the abundance of Akkermansiaceae was comparable between the normal-control and PBEH-H groups. In contrast, the control group exhibited a higher abundance of Akkermansiaceae than both groups (Figure S3A). Additionally, the abundance of Bifidobacteriaceae was lower in the control group compared to the normal-control group. However, it was elevated in both the PBEH and positive-control groups relative to the control. Notably, the PBEH-H group displayed the highest level of Bifidobacteriaceae among all groups. Furthermore, the control group demonstrated a decreasing trend of Lachnospiraceae compared to the normal-control group, with consistently lower values observed in all groups treated with PBEH (Figure S3A).

**Figure 4:**
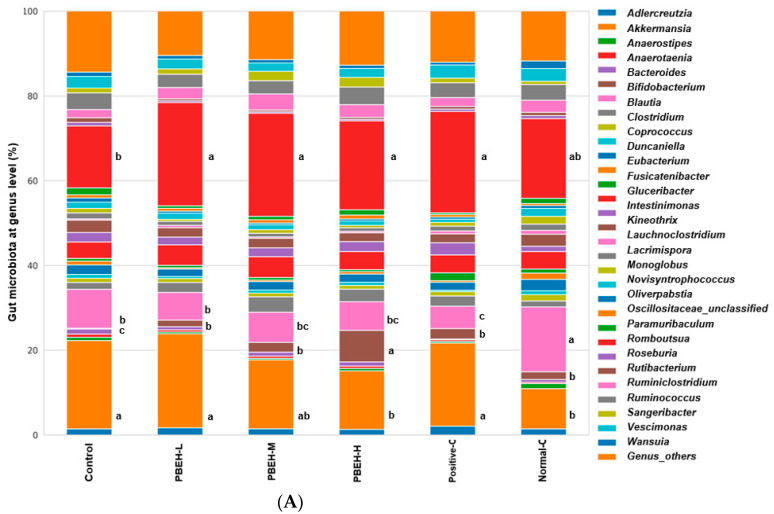

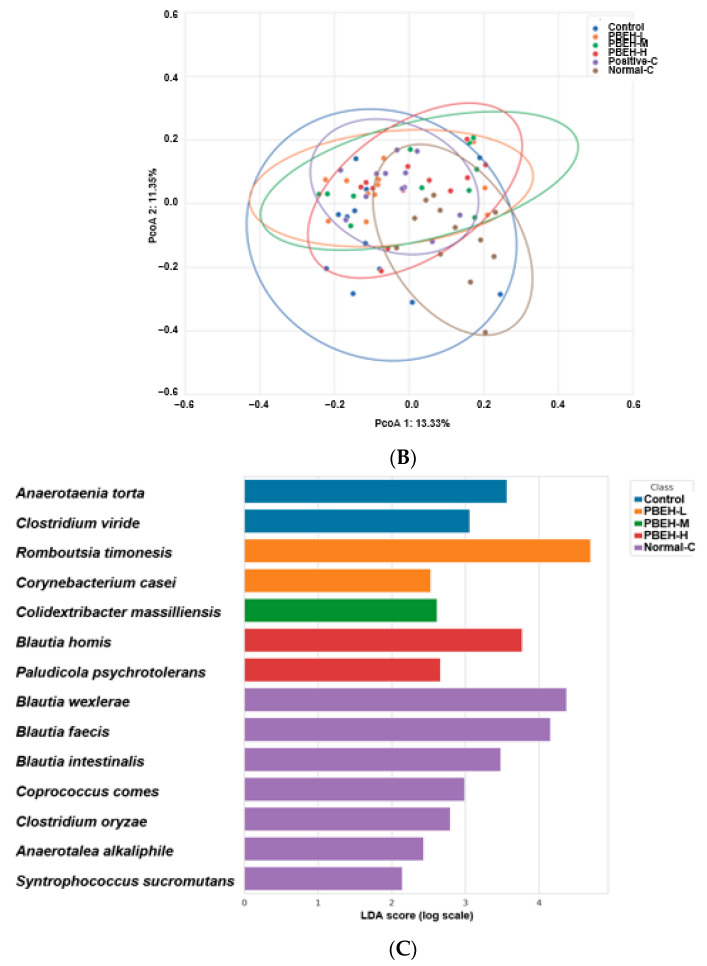

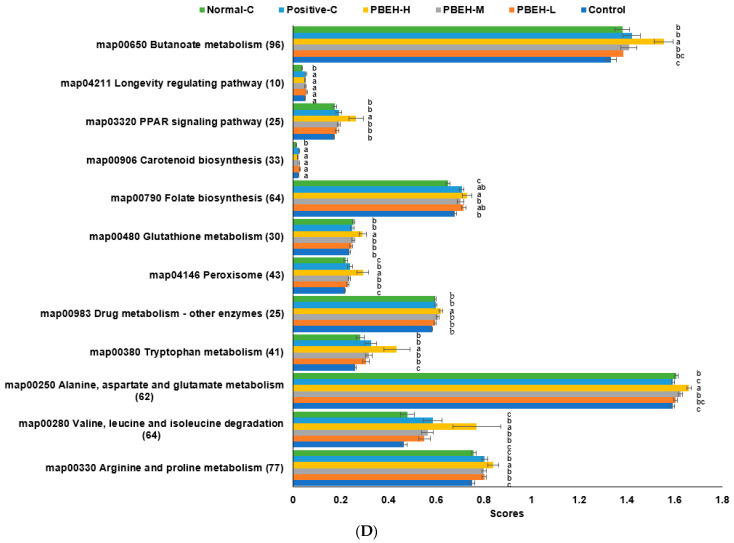
Gut microbiota community. (**A**) Gut microbiota in the cecum at the genus level; (**B**) β-diversity of the gut microbiota; (**C**) Gut microbiota with significant LDA scores in each group; (**D**) Metabolic function of gut microbiota analyzed by Picrust2. Bars and error bars are presented as means ± standard deviation (*n* = 10). Different superscripts (a, b, c) denote significant differences using Tukey’s test among the groups at *p* < 0.0001.

The trends observed for Bifidobacteriaceae, Akkermansiaceae, and Lachnospiraceae at the genus level were consistent with those observed at the family level (Figure 4A). Specifically, *Akkermansia* increased, and *Bifidobacterium* decreased in the control group compared to the normal-control group. Conversely, there was a decrease in *Akkermansia* and an increase in *Bifidobacterium* in the PBEH-H group compared to the control group. Moreover, *Blautia* exhibited a lower abundance in the control group than the normal-control group, while the PBEH groups displayed similar levels to the control group. Interestingly, the positive-control group contained a lower abundance of *Blautia* than the control group (Figure 4A). Furthermore, *Romboutsia* at the genus level showed a decreasing trend in the control group compared to the normal-control group, with an elevated abundance observed in the PBEH groups relative to the control group (Figure 4A).

The Chao1 and Shannon indices (α-diversity index) did not reveal statistically significant differences among the control, normal-control, positive-control, and PBEH groups (Figure S3B). However, β-diversity, representing the distribution of intestinal microorganisms across groups, exhibited significant differences. Specifically, it was significantly different in the PBEH-L (*p* = 0.009), PBEH-M (*p* = 0.002), PBEH-H (*p* = 0.009), positive-control (*p* = 0.001), and normal-control (*p* = 0.002) groups compared to the control group. Furthermore, the β-diversity of PBEH-H differed from that of PBEH-L (*p* = 0.044), normal-control (*p* < 0.001), and positive-control (*p* = 0.027) groups. Additionally, it was also different in PBEH m compared to normal-control (*p* < 0.001) and positive-control (*p* = 0.044; Figure 4B). Linear discriminant analysis effect size (LEfSe) indicated the most abundant genera in each group, represented using the linear discriminant analysis (LDA) scores (Figure 4C). The control group exhibited high LDA scores for *Anaerotenia torta* and *Clostridium viride*, the PBEH-L group showed high LDA scores for *Romboutsia timonensis* and *Corynebacterium casei*, the PBEH-M group had a high LDA score for *Colidextribacter massiliensis,* the PBEH-H group had high LDA scores for *Blautia hominis* and *Paludicola psychrotolerans*. The normal-control group showed high LDA scores for *Blautia wexlerae, Blautia faecis, Blautia intestinalis, Coprococcus comes, Anaerotalea alkaliphila*, and *Syntrophococcus sucromutans* (Figure 4C).

The PICRUSt analysis revealed minimal differences in microbial function between the control and normal-control groups. However, compared to the control group, the PBEH-H group demonstrated the activation of mechanisms involved in xenobiotic metabolism (Figure 4D). Amino acid metabolism, butanoate metabolism, glutathione metabolism involved in antioxidant defense, and synthesis of folate and carotenoids associated with antioxidant properties were enhanced in the PBEH-H group (Figure 4D). Thus, metagenome function in the PBEH-H group mainly improved antioxidant defense in an immunodeficient animal model.

## 4. Discussion

Our study aimed to investigate the effects of PBEH consumption on the immune function and gut microbiota composition in a CTX-induced immunodeficient animal model. We observed significant alterations in the immune function in CTX-induced immunodeficient rats, including reduced NK cell activity, splenocyte proliferation in response to LPS, decreased cytokine production leading to a decreased Th1/Th2 ratio, and structural damage to the spleen, the primary immune organ. Hepatic and splenocyte lipid peroxide contents were higher in the control group than in the normal-control group, and PBEH dose-dependently decreased them. These effects were associated with alterations in the cecal microbiome function. CTX administration induced gut microbiota dysbiosis, ameliorated using PBEH supplementation, particularly by improving antioxidant defense mechanisms. Importantly, PBEH intake prevented immune dysfunction by enhancing immunodeficient-related responses without eliciting autoimmune responses induced using CTX. Furthermore, PBEH intake promoted eubiosis, thereby enhancing antioxidant activity. These findings suggest the potential therapeutic implications of PBEH for improving cell-mediated immune deficiency.

CTX, an alkylating agent widely used in treating various hematological malignancies such as lymphomas, multiple myeloma, and leukemia, is known for its immunosuppressive effects [19]. This drug inhibits rapid cell division, including that of immune cells like WBC, thereby interfering with T-cell function—an essential component of the adaptive immune system. Consequently, this interference potentially compromises the body’s ability to mount effective immune responses against pathogens and cancer cells. Additionally, CTX diminishes B-cell activity, reducing antibody production [19,20]. Assessment of immune function, crucial for understanding the impact of CTX administration, involves evaluating key immune parameters such as NK cell activity [21] and splenocyte proliferation [22]. Our study revealed that immune-deficient control rats exhibited significantly lower total WBC and lymphocyte counts than normal-control rats, underscoring the impact of CTX on immune cell populations. Furthermore, the spleen index—an indicator of both innate and adaptive immunity—was markedly reduced in the control group. Additionally, CTX administration diminished NK cell activity and impaired splenocyte proliferation in response to the LPS treatment. These findings collectively indicated that CTX induced immune deficiency, highlighting its immunosuppressive effects in our study model.

Furthermore, alterations in Th1 and Th2 cytokine production and the Th1/Th2 ratio are essential for understanding the immunological changes associated with immune suppression [23]. The balance between the Th1 and Th2 cytokines is crucial for orchestrating effective immune responses, and disruptions in this balance can lead to immune dysregulation and increased susceptibility to infections or autoimmune diseases [23]. The Th1/Th2 ratio, which reflects the balance between Th1 and Th2 cytokine production, is critical for maintaining immune homeostasis [6]. A higher Th1/Th2 ratio is associated with a predominance of cell-mediated immunity, while a lower ratio indicates a shift towards humoral immunity [24]. In the present study, the IFN-γ in Th1 cytokines was lower in the control than in the normal-control, and PBEH-H increased it as much as in the positive-control and normal-control groups. Serum IL-4 concentrations, representing Th1 cells, were higher in the control group than in the normal-control group and similar in the control and PBEH groups. Interestingly, serum IL-4 concentrations were elevated in the positive-control group, whereas serum IL-6 concentrations were higher in the PBEH m group compared to the control. Moreover, the Th1/Th2 ratio was significantly lower in the control group compared to the normal-control group. PBEH-H supplementation increased the ratio to levels similar to those observed in the normal-control group. These findings suggest that CTX-induced immunodeficiency may be linked to a weakened cell-mediated immune response. However, supplementation with PBEH-H appears to enhance immune responses via cell-mediated immunity. Given the broad impact of immunodeficiency on human health, there is considerable interest in exploring natural products and their components for their potential immunomodulatory effects. Previous research has investigated natural compounds such as curcumin, ginseng, and flavonoids for their ability to modulate immune responses [25,26,27]. Ginseng and its bioactive components have demonstrated immune-stimulating properties in animal models of immunosuppression induced using CTX, including increased activity of NK cells and enhanced antibody production [9,28,29]. Our study designated red ginseng extract (100 mg/day) as the positive-control. While some amino acids and peptides have also shown promise in alleviating immunosuppression [30], the potential immunomodulatory effects of PBEH have not been thoroughly investigated [31]. In our investigation, PBEH supplementation resulted in a significant but slight increase in the immune parameters compared to the control group, with values similar to those observed with ginseng extract (positive-control). Specifically, PBEH-H supplementation was associated with a slightly higher spleen index than the control group. Furthermore, PBEH-H supplementation elevated NK cell activity and splenocyte proliferation in response to LPS stimulation to levels similar to those observed in the normal-control group. Notably, NK cell activity in the PBEH m group was superior to the positive-control group. These findings suggest that PBEH-H exhibits similar or superior immune-enhancing effects to red ginseng extract. This well-known immunomodulator boosts cell-mediated and humoral immunity against various health conditions [10].

In addition to its immunosuppressive effects, CTX induces various metabolic alterations, including weight loss and hepatic and renal dysfunction [32,33]. In our study, CTX administration suppressed weight gain during the experimental period, a phenomenon mitigated to some extent by PBEH supplementation. While PBEH protected against the decrease in weight gain compared to the control group, the effect was insufficient to ensure weight equivalent to the normal-control group, suggesting a potential link between PBEH intake and regulation of food intake. Consistent with the known hepatotoxic effects of CTX, we observed evidence of liver damage in the immune-suppressed rats, as indicated by elevated serum ALT and AST concentrations and lipid peroxide content in the liver. However, supplementation with PBEH effectively inhibited the increase in ALT and AST levels and hepatic lipid peroxide contents, indicating its hepatoprotective effects. Specifically, the PBEH m and positive-control groups exhibited serum ALT concentrations at comparable levels in the normal-control group. In the present study, immunodeficiency increased lipid peroxide contents in the liver and splenocytes, consistent with previous studies that indicated that immune deficiency increases oxidative stress [34,35]. PBEH intake reduced hepatic and splenocyte lipid peroxide contents. These findings suggest that PBEH supplementation mitigates the adverse effects of CTX-induced liver damage by decreasing lipid peroxides.

The gut microbiota is pivotal in shaping the immune system, influencing innate and adaptive immunity [6,36]. This intricate relationship between the gut microbiota and immunity is essential for maintaining immune homeostasis, antioxidant defense, defending against pathogens, and regulating inflammatory responses [6]. Central to this relationship is the gut barrier function, which prevents immune activation and inflammation in response to gut microbial antigens [37]. Our study revealed that immunodeficient rats exhibited alterations in intestinal morphology compared to the normal-control group. Specifically, they displayed shorter villi length, crypt height, and goblet cells producing mucin and higher villi width, potentially indicative of a compromised intestinal barrier function [37]. Remarkably, supplementation with PBEH-H improved intestinal morphology, restoring it to similar levels observed in the normal-control group. The gut microbiota also contributes significantly to the induction of immune tolerance, thereby preventing inappropriate immune responses against harmless dietary antigens and commensal bacteria [38]. This process involves generating regulatory T cells (Tregs) and suppressing pro-inflammatory immune pathways, thus maintaining immune homeostasis and averting autoimmunity [39]. The present study demonstrated that the Th1/Th2 ratio was lower in the control group than in the normal-control group, and PBEH supplementation increased this ratio. The result suggests a bolstering of immunity without the simultaneous activation of autoimmunity. Additionally, immunodeficient rats exhibited increased levels of *Akkermansia muciniphila*, which is known for its role in mucin degradation and production. However, despite this increase, there was no elevation in goblet cells, indicating a potential dysbiosis. Importantly, the PBEH-H supplementation maintained goblet cell levels despite decreased *Akkermansia* while also elevating *Bifidobacterium*, thereby improving innate immunity. These findings suggest that immunodeficiency may lead to dysbiosis and that PBEH supplementation could serve as a preventive measure to enhance immunity by restoring gut microbiota balance.

## 5. Conclusions

PBEH intake prevented immune dysfunction and oxidative stress by activating immune responses without stimulating the autoimmune responses induced using CTX. Moreover, PBEH improved gut microbiota composition, promoting eubiosis and preserving the gut barrier function. These results suggest that PBEH may have therapeutic implications for immune-deficient states by enhancing immune function, maintaining gut microbiota homeostasis, and improving antioxidant defense mechanisms. Furthermore, our study highlights the importance of understanding the immunological changes associated with immune suppression, including alterations in Th1 and Th2 cytokine production and the Th1/Th2 ratio. The PBEH supplementation restored the Th1/Th2 balance, enhancing cell-mediated immunity without exacerbating humoral immunity and decreasing lipid peroxide contents by activating antioxidant defense in metagenome function. Overall, our study provides valuable insights into the therapeutic potential of PBEH in immune deficiency. Further research is warranted to elucidate the underlying mechanisms and clinical implications of PBEH supplementation in immune-related disorders, indicating a promising avenue for a clinical investigation.

## Figures and Tables

**Table 1:** Energy metabolism, immunity, and liver damage-related parameters in immunodeficient rats.

	Control	PBEH-L	PBEH-M	PBEH-H	Positive-Control	Normal-Control
Body weight gain (g)	175 ± 20.3 ^c^	203 ± 19.5 ^b^	185 ± 20.4 ^bc^	194 ± 21.4 ^b^	179 ± 24 ^c^	222 ± 19.7 ^a^
Food intake (g/day)	24 ± 1.7 ^b^	26 ± 0.9 ^a^	25.1 ± 0.6 ^ab^	25 ± 0.8 ^ab^	24.1 ± 1.2 ^b^	26.5 ± 1.1 ^a^
Spleen index	1.89 ± 0.44 ^c^	2.07 ± 0.3 ^bc^	2.02 ± 0.28 ^bc^	2.16 ± 0.36 ^b^	2.1 ± 0.42 ^bc^	2.75 ± 0.49 ^a^
Thymus index	1.52 ± 0.48 ^b^	1.5 ± 0.49 ^b^	1.38 ± 0.17 ^b^	1.4 ± 0.25 ^b^	1.41 ± 0.33 ^b^	1.84 ± 0.29 ^a^
Hepatic lipid peroxide contents (nmol/mg of tissue)	0.63 ± 0.05 ^a^	0.58 ± 0.06 ^ab^	0.54 ± 0.06 ^b^	0.47 ± 0.05 ^c^	0.54 ± 0.05 ^b^	0.48 ± 0.05 ^c^
Serum ALT (IU/L)	18.8 ± 0.71 ^a^	16.1 ± 1.48 ^b^	12.4 ± 0.57 ^c^	16 ± 0.74 ^b^	13 ± 0.93 ^c^	12 ± 1.52 ^c^
Serum AST (IU/L)	28 ± 0.52 ^a^	27.7 ± 0.7 ^a^	26.1 ± 0.54 ^b^	27.1 ± 1.38 ^ab^	28.4 ± 0.88 ^a^	22.6 ± 0.79 ^c^

The values are presented as means ± standard deviation (*n* = 10). Different superscripts (a, b, c) denote significant group differences using Tukey’s test at *p* < 0.05.

**Table 2:** White blood cell counts and their components in immunodeficient rats.

	Control	PBEH-L	PBEH-M	PBEH-H	Positive-Control	Normal-Control
WBC (10^9^/L)	2.82 ± 0.55 ^b^	3.11 ± 1.01 ^b^	3 ± 0.51 ^b^	3.13 ± 0.73 ^b^	3.18 ± 0.95 ^b^	7.05 ± 1.86 ^a^
Lymphocyte (10^9^/L)	1.51 ± 0.54 ^c^	1.88 ± 0.61 ^bc^	1.78 ± 0.45 ^bc^	1.91 ± 0.5 ^b^	1.93 ± 0.73 ^b^	5.18 ± 1.55 ^a^
Granulocytes (10^9^/L)	1.17 ± 0.22 ^b^	1.1 ± 0.46 ^b^	1.09 ± 0.25 ^b^	1.08 ± 0.25 ^b^	1.12 ± 0.31 ^b^	1.65 ± 0.53 ^a^
Mid white blood cell (10^9^/L)	0.13 ± 0.04 ^b^	0.13 ± 0.03 ^b^	0.13 ± 0.03 ^b^	0.13 ± 0.04 ^b^	0.12 ± 0.03 ^b^	0.22 ± 0.08 ^a^

The values are presented as means ± standard deviation (*n* = 10). Different superscripts (a, b, c) denote significant group differences using Tukey’s test at *p* < 0.05.

**Table 3:** Splenocyte proliferation and natural killer cells in isolated splenocytes.

	Control	PBEH-L	PBEH-M	PBEH-H	Positive-Control	Normal-Control
Splenocyte proliferation without LPS induction (%)	80.6 ± 19.3	84.2 ± 17.4	91.7 ± 34.7	95.9 ± 24.6	98.2 ± 11	100 ± 27.1
Splenocyte proliferation with LPS induction (10 ng/mL)	72.4 ± 11.5 ^b^	79.1 ± 7.5 ^b^	75.2 ± 11.5 ^b^	89.6 ± 7.6 ^a^	87.4 ± 10 ^a^	100 ± 16.7 ^a^
Lipid peroxide contents in splenocytes	0.93 ± 0.08 ^a^	0.81 ± 0.08 ^b^	0.76 ± 0.07 ^b^	0.64 ± 0.06 ^c^	0.82 ± 0.08 ^b^	0.76 ± 0.08 ^b^
Natural killer cell activity(%)	51.4 ± 9.1 ^c^	81.7 ± 11.5 ^b^	73 ± 15.5 ^b^	93.6 ± 15 ^a^	76.6 ± 7.6 ^b^	100 ± 14.4 ^a^

The values are presented as means ± standard deviation (*n* = 10). Different superscripts (a, b, c) denote significant group differences using Tukey’s test at *p* < 0.05. LPS, lipopolysaccharide.

**Table 4:** Serum concentrations of immunoglobulins and cytokines in immunodeficient rats.

	Control	PBEH-L	PBEH-M	PBEH-H	Positive-Control	Normal-Control
IgA (μg/mL)	91.7 ± 5.64 ^c^	95.7 ± 5.14 ^c^	106.4 ± 6.32 ^ab^	103.7 ± 6.14 ^b^	102.1 ± 3.95 ^b^	114.6 ± 5.47 ^a^
IgG (mg/mL)	4.85 ± 0.34 ^c^	4.97 ± 0.41 ^c^	5.61 ± 0.38 ^b^	6.03 ± 0.38 ^ab^	5.78 ± 0.43 ^ab^	6.38 ± 0.39 ^a^
IgM (mg/mL)	0.72 ± 0.04 ^b^	0.78 ± 0.05 ^b^	0.87 ± 0.04 ^a^	0.89 ± 0.06 ^a^	0.84 ± 0.08 ^a^	0.91 ± 0.07 ^a^
IL-1β (pg/mL)	17.6 ± 0.92 ^a^	17.6 ± 1.13 ^a^	15.7 ± 0.68 ^b^	15.7 ± 0.97 ^b^	15 ± 0.84 ^b^	18.5 ± 1.15 ^a^
IL-4 (pg/mL)	29 ± 1.55 ^a^	27.5 ± 1.69 ^ab^	29.8 ± 1.67 ^a^	29.3 ± 1.43 ^ab^	31.3 ± 2 ^a^	26.6 ± 1.38 ^b^
IL-6 (pg/mL)	11.4 ± 0.9 ^b^	12.7 ± 1.01 ^ab^	13.4 ± 1.1 ^a^	12.6 ± 1.3 ^ab^	11.6 ± 0.8 ^b^	9.4 ± 0.99 ^c^
IFN-γ (pg/mL)	13.5 ± 0.48 ^b^	14.5 ± 0.39 ^ab^	14.2 ± 0.54 ^ab^	15.1 ± 0.73 ^a^	14.8 ± 0.72 ^a^	14.8 ± 0.42 ^a^
Th1/Th2 ratio	0.46 ± 0.02 ^c^	0.53 ± 0.02 ^b^	0.48 ± 0.02 ^c^	0.52 ± 0.03 ^b^	0.47 ± 0.03 ^c^	0.56 ± 0.02 ^a^

The values are presented as means ± standard deviation (*n* = 10). Different superscripts (a, b, c) denote significant group differences using Tukey’s test at *p* < 0.05.

## Data Availability

The data presented in this study are available upon request from the corresponding author.

## Supplementary Materials

The following supporting information can be downloaded at: https://www.mdpi.com/article/10.3390/antiox13040476/s1, Figure S1: Body weight changes during the experimental periods; Figure S2: Gut microbiota in the cecum at the family level; Figure S3: The morphology of large intestines; Table S1: Amino acid composition in porcine brain enzyme hydrolysates.

## Abbreviations

Porcine brain enzyme hydrolysate, PBEH; cyclophosphamide, CTX; lipopolysaccharide, LPS; central nervous system, CNS; autonomic nervous system, ANS; ethylenediaminetetraacetic acid, EDTA; natural killer, NK; white blood cell, WBC; Interferon-γ, IFN-γ; interleukin, IL; enzyme-linked immunosorbent assay, ELISA; alanine aminotransferase, ALT; aspartate aminotransferase, AST; lipopolysaccharides, LPS; lactate dehydrogenase, LDH; perchloric acid, PAS; short-chain fatty acid, SCFA; polymerase chain reaction, PCR; phylogenetic investigation of communities by reconstruction of unobserved states 2, PICRUSt2; Kyoto encyclopedia of genes and genomes, KEGG; KEGG Orthologues, KO; one-way analysis of variance, ANOVA.
