# Supplementary material for: Porcine Brain Enzyme Hydrolysate Enhances Immune Function and Antioxidant Defense via Modulation of Gut Microbiota in a Cyclophosphamide-Induced Immunodeficiency Model
Source: Antioxidants (Basel). 2024 Apr 17;13(4):476. doi: 10.3390/antiox13040476 (PMC11047735; doi:10.3390/antiox13040476)
Supplement: Supplementary file 1 [file antioxidants-13-00476-s001.zip › antioxidants-2930617-supplementary.pdf]

Supplementary figures

Figure S1. Body weight changes during the experimental periods

* Significantly different among the groups at each time point at <0.05.

Figure S2. Gut microbiota in the cecum at the family level

Figure S3. The morphology of large intestines

A. Large intestine villi length and width and crypt height
B. Hematoxylin-eosin staining in the large intestines
C. Goblet cell contents in the large intestines
D. PAS staining in the large intestines

Bars and error bars represent the means ± standard deviations (n = 10).

[a,b,c] Different superscript letters on the bars indicated a significant difference among the groups by Tukey test at $P<0.05$.

Fig. S1

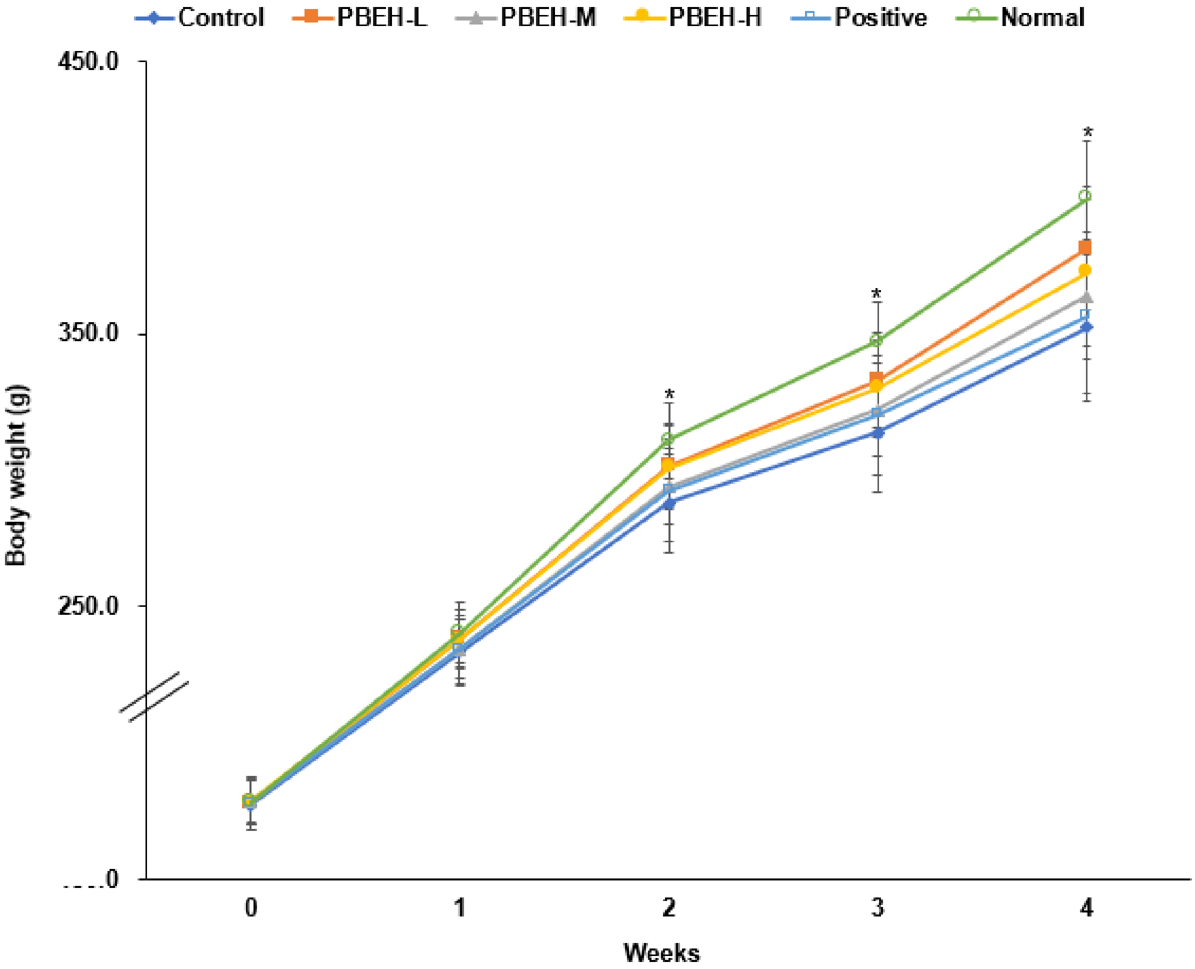

Control
PBEH-L
PBEH-M
PBEH-H
Positive
Normal
450.0
350.0
250.0
Body weight (g)
0
1
2
3
4
Weeks
*
*
*

Fig. S2A

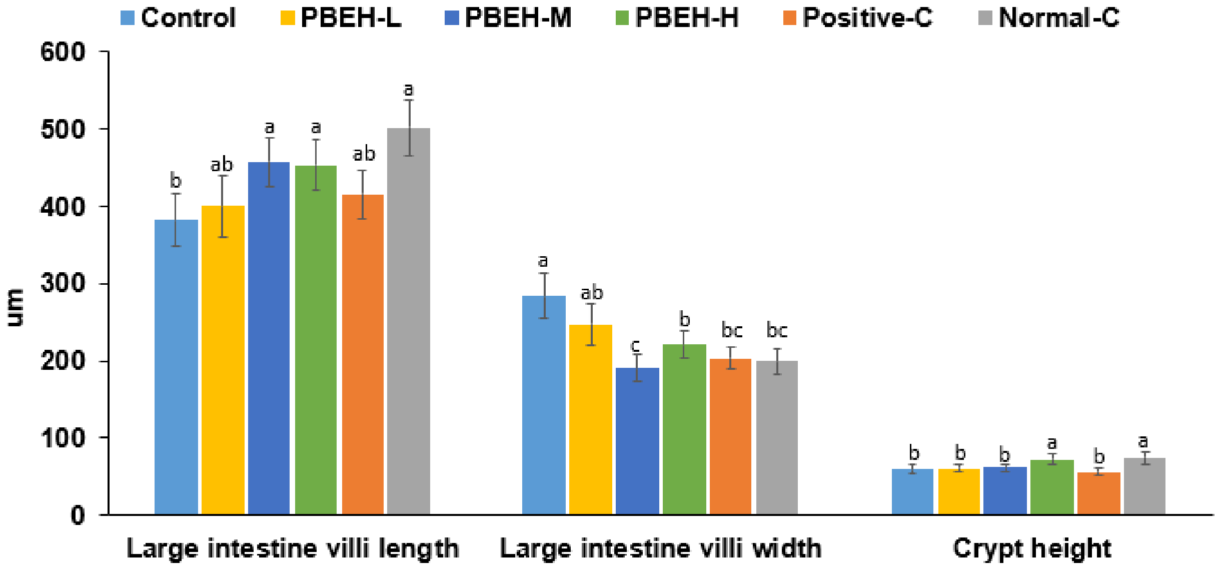

Fig. S2B

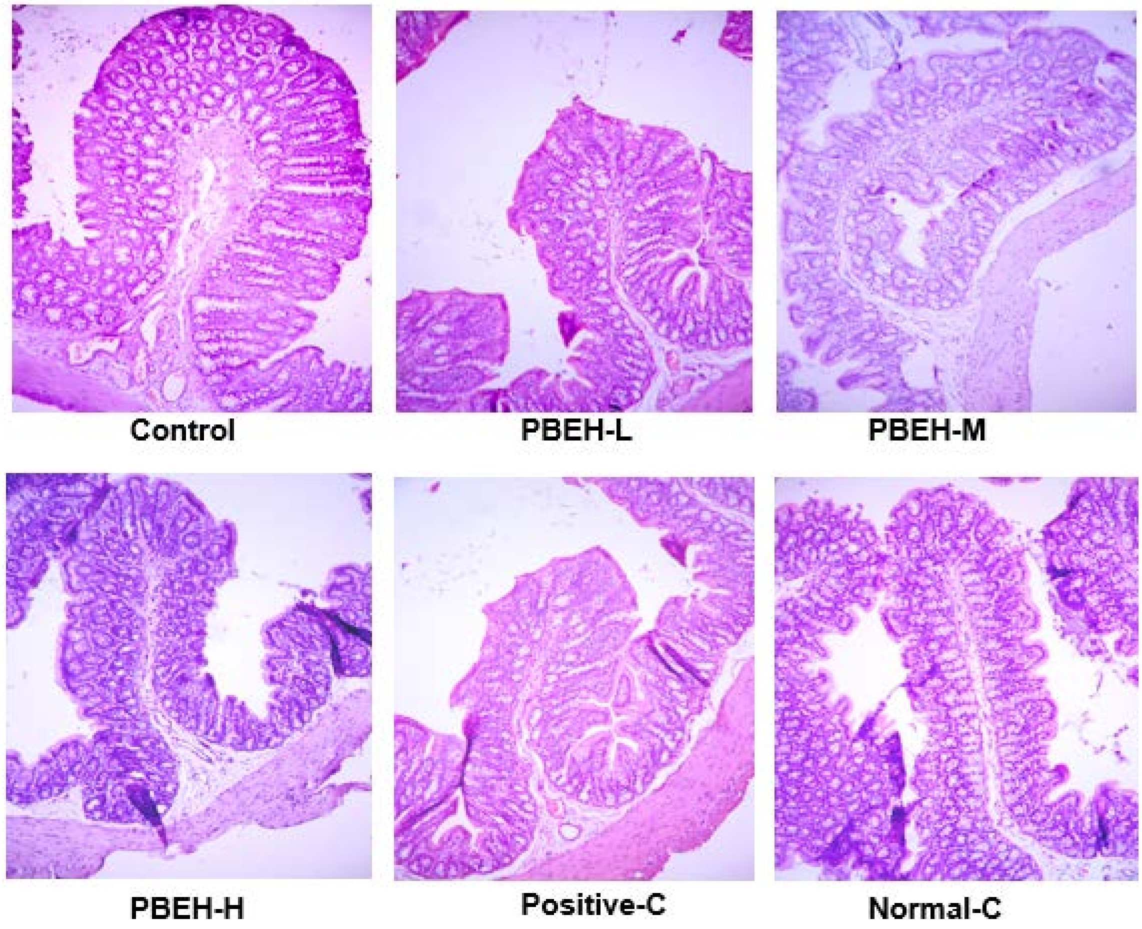

Fig. S2C

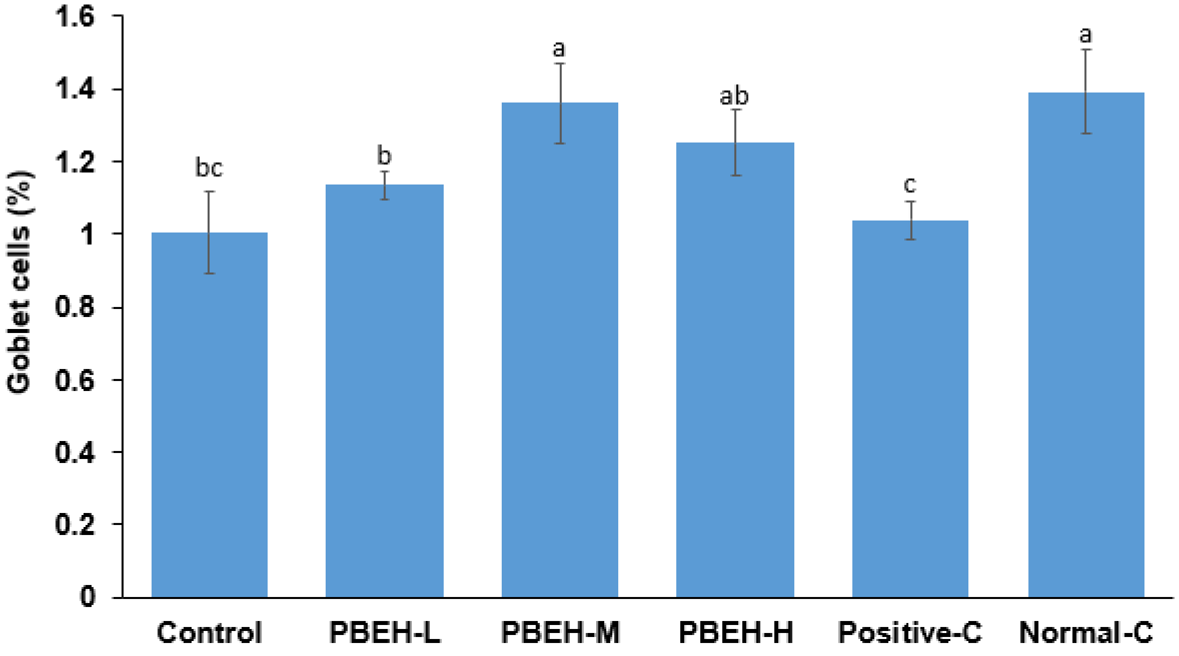

Fig. S2D

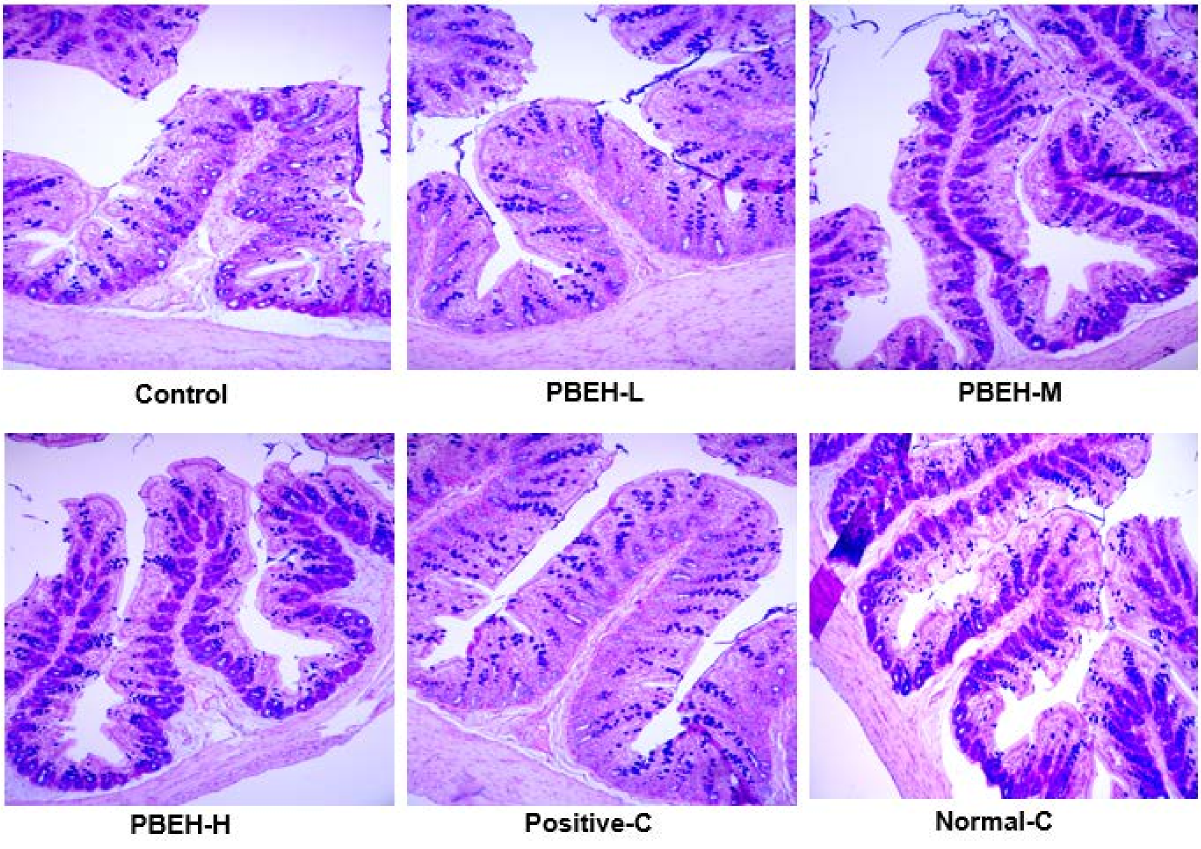

Fig. S3A

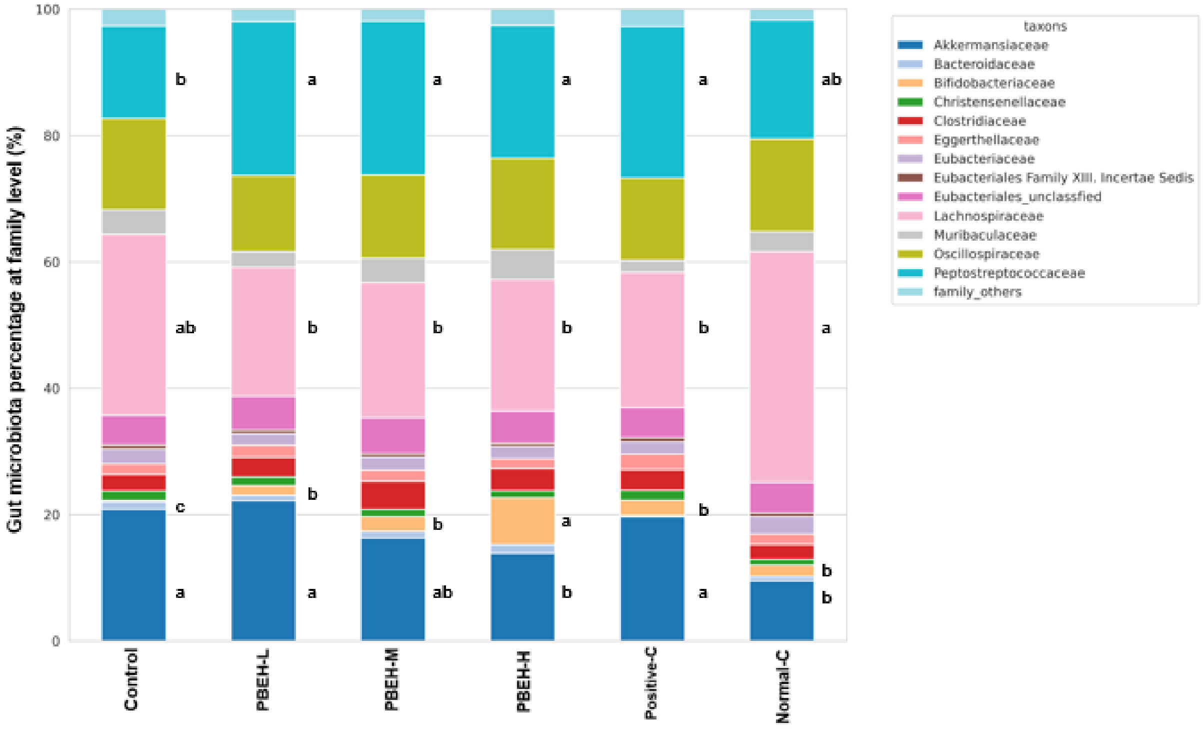

Fig. S3B

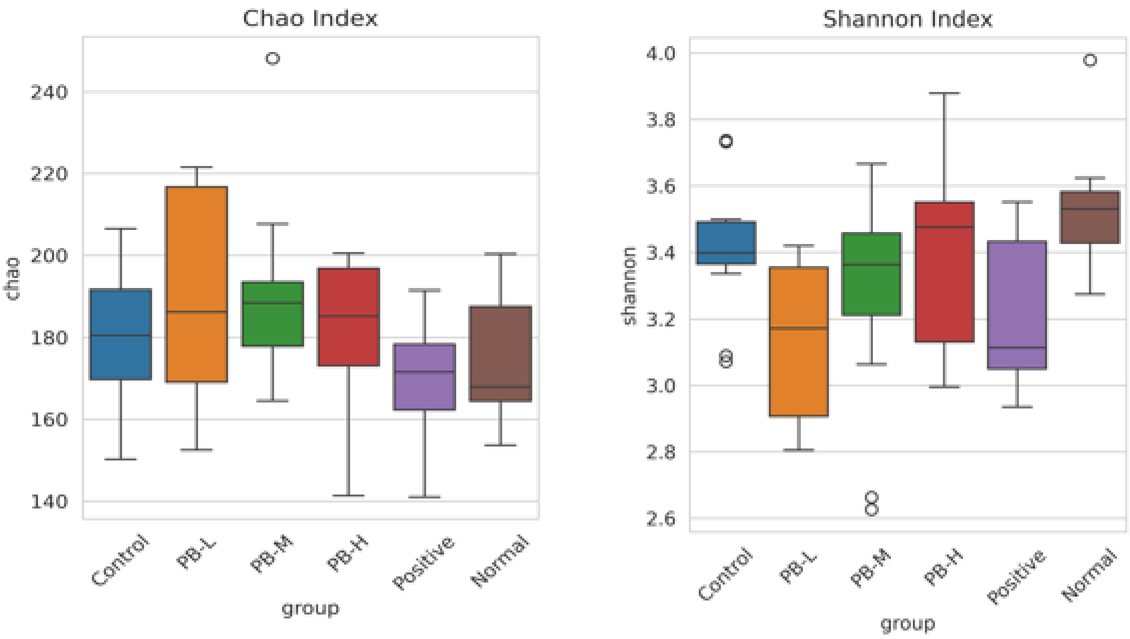

Supplementary Table S1. Amino acid composition in porcine brain enzyme hydrolysates

Amino acids	PBEH (mg/mL)	Amino acids	PBEH (mg/mL)
ARG	2.37±0.12	ALA	0.65±0.04
LUE	2.33±0.063	SER	0.56±0.02
LYS	2.15±0.09	ASP	0.49±0.02
PHE	1.64±0.03	PRO	0.47±0.03
ILU	0.86±0.02	HIS	0.46±0.03
MET	0.77±0.04	TRE	0.36±0.01
VAL	0.79±0.03	GLY	0.19±0.02
GLU	0.68±0.02		
